# Research on Asymmetric Hysteresis Modeling and Compensation of Piezoelectric Actuators with PMPI Model

**DOI:** 10.3390/mi11040357

**Published:** 2020-03-30

**Authors:** Wen Wang, Jian Wang, Zhanfeng Chen, Ruijin Wang, Keqing Lu, Zhiqian Sang, Bingfeng Ju

**Affiliations:** 1 School of Mechanical Engineering, Hangzhou Dianzi University, Hangzhou 310018, China; wangwn@hdu.edu.cn (W.W.); wangjian_0826@126.com (J.W.); wangrjcn@163.com (R.W.); lkq@hdu.edu.cn (K.L.); sang@hdu.edu.cn (Z.S.); 2 State Key Laboratory of Fluid Power Transmission and Control, Zhejiang University, Hangzhou 310027, China; mbfju@zju.edu.cn

**Keywords:** piezoelectric actuators (PEAs), asymmetric hysteresis, Prandtl–Ishlinskii (PI) model, polynomial-modified PI (PMPI) model, feedforward hysteresis compensation

## Abstract

Because of fast frequency response, high stiffness, and displacement resolution, the piezoelectric actuators (PEAs) are widely used in micro/nano driving field. However, the hysteresis nonlinearity behavior of the PEAs affects seriously the further improvement of manufacturing accuracy. In this paper, we focus on the modeling of asymmetric hysteresis behavior and compensation of PEAs. First, a polynomial-modified Prandtl–Ishlinskii (PMPI) model is proposed for the asymmetric hysteresis behavior. Compared with classical Prandtl–Ishlinskii (PI) model, the PMPI model can be used to describe both symmetric and asymmetric hysteresis. Then, the congruency property of PMPI model is analyzed and verified. Next, based on the PMPI model, the inverse model (I-M) compensator is designed for hysteresis compensation. The stability of the I-M compensator is analyzed. Finally, the simulation and experiment are carried out to verify the accuracy of the PMPI model and the I-M compensator. The results implied that the PMPI model can effectively describe the asymmetric hysteresis, and the I-M compensator can well suppress the hysteresis characteristics of PEAs.

## 1. Introduction

With the growth of semiconductor and precision manufacturing industries, the positioning accuracy of micro/nano scale is highly required [1,2,3,4]. Because of the advantages of fast frequency response, high stiffness and displacement resolution, piezoelectric actuators (PEAs) based on inverse piezoelectric effect have gradually become one of the most widely used smart material actuators. In addition, PEAs are popularly applied as the actuators in micro/nano scale measurement, micro-electro-mechanical systems (MEMS), flexible electronics manufacturing, and biomedical engineering. However, strong nonlinear hysteresis of piezoelectric materials makes the output of PEAs difficult to predict, and the positioning accuracy and stability of the system are low [5,6]. Specifically, scholars provided plenty of mathematical models to describe the symmetric hysteresis. However, effective mathematical model to describe the asymmetric hysteresis is still lacking, as the asymmetric hysteresis is a more common hysteresis nonlinear phenomenon. Therefore, it is worth exploring the modeling of asymmetric hysteresis behavior and using it to compensate piezoelectric actuators.

To eliminate the influence of hysteresis on accuracy and stability of the system, scholars have proposed numerous hysteresis models and controllers. These hysteresis models can be divided into two categories [7]: mechanistic model and phenomenological model. The former are based on basic physical principles, and derived by energy-displacement or stress–strain methods. Among them, the famous models are Jiles–Atherton model [8,9] and Domain Wall model [10]. The latter uses directly mathematical means to characterize the hysteresis, and ignore the physical meaning behind the hysteresis phenomenon. Because of the high accuracy and flexibility, the phenomenological model is more popular in hysteresis modeling. The phenomenological models include Preisach model [11,12], polynomial model [13,14], Bouc–Wen model [15,16], Duhem model [17,18], neural network model [19,20], Prandtl–Ishlinskii (PI) model [21,22,23], and etc. Among them, because of simple expression and analytical inverse model, the PI model is the most widely used in hysteresis modeling and compensation. However, the PI model utilizes weighted superposition of the Play operators to describe hysteresis nonlinearity; the Play operator is a basic component of the PI model. Therefore, the symmetric structure of the Play operator determines that PI model can only describe symmetric hysteresis.

**Figure 1:**
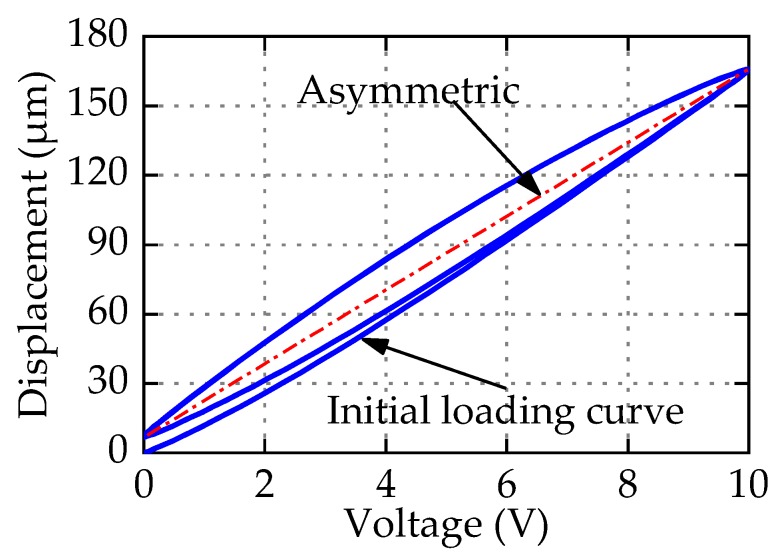
Asymmetric hysteresis loops of piezoelectric actuators (PEAs).

Actually, PEAs have slight or severe asymmetric characteristics, as shown in Figure 1. When the application scope of PEAs scales down to micro/nano-meter levels, the gap between symmetric and asymmetric hysteresis modeling approaches results in positioning error. To describe asymmetric hysteresis, scholars [24,25,26] have tried to modify the PI model, and designed the corresponding controller. Kuhnen [24] presented the dead-zone operator and the modified PI (Ku-PI) model to describe the asymmetric hysteresis. Janaideh et al. [25] provided the generalized PI (GPI) model based on a nonlinear play operator. Different envelope functions make the GPI model have flexible ascending and descending edge. Hence, the GPI model can describe accurately the complex hysteresis phenomenon. By combining memoryless polynomial with PI model, Gu [26] proposed a new modified PI (Gu-PI) model to depict asymmetric hysteresis. These modified PI models and corresponding controller play a good role in the micro/nano positioning compensation. However, these models still have some limitations. The parameter identification of Ku-PI model is complex, which increases the difficulty of compensator design; GPI model is flexible and accurate, but choosing envelope function still depends on the experience and recursive debugging; Gu-PI model is simple in structure and easy to identify, and has good performance with one-side Play operator when the initial loading curve is not considered. But we have tried to extend the application of Gu-PI model with two-side Play operator when the initial loading curve is considered, and its effect is poor. The reason is that the Play operator lacks accuracy in describing displacements near the zero voltage.

In this paper, a new polynomial-modified PI (PMPI) model is proposed to describe the asymmetric hysteresis of PEAs. Compared with PI model, the most important innovation of PMPI model is the introduction of Modified-Play (M-Play) operator and memoryless polynomial. M-Play operator replaces Play operator as the basic operator in the PMPI model. The memoryless polynomial enables PMPI model to describe the asymmetric hysteresis. The shape of asymmetric hysteresis is determined by both weighted M-Play operators and memoryless polynomial. The main advantages of PMPI model are as following: (1) Whether the initial loading curve is considered or not, in PMPI model, the displacement near zero voltage can be described flexibly by M-Play operator. (2) The inverse of PMPI model can be derived directly from PI model. The feedforward compensation of hysteresis in real-time application can be carried out. To validate the proposed PMPI model and I-M compensator, simulation and experiment are conducted on a piezoelectric micromotion platform.

This paper is organized as follows: Section 2 introduces PMPI model and examines the congruency property. Section 3 develops an I-M compensator and analyzes its stability. Section 4 verifies the PMPI model and I-M compensator on a piezoelectric micromotion platform. Section 5 concludes this paper.

## 2. Polynomial-Modified Prandtl–Ishlinskii Model

Before introducing the proposed PMPI model, it is necessary to review the PI model in brief.

### 2.1. Prandtl–Ishlinskii Model

**Figure 2:**
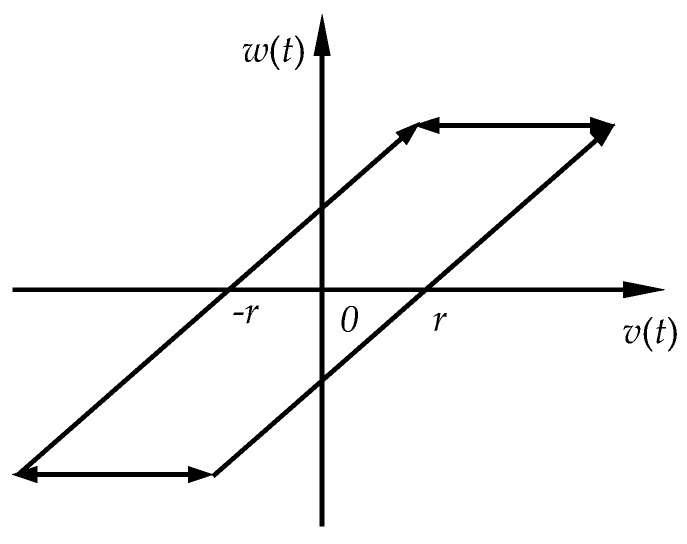
The input–output relationship of the Play operator.

The PI model utilizes weighted superposition of the Play operators to describe hysteresis nonlinearity, so the Play operator is the basic component of PI model. Without special description in the following paper, the Play operator refers to two-side Play operator. For any piecewise monotonic input signal *v*(*t*) ∈ *C_m_*(0 *t_N_*), the output *w*(*t*) = *F_r_*(*v*)(*t*) of the Play operator with threshold *r* is defined as

(1) $$\begin{array}{l} w(0)=F_{r}(v)(0)=f_{r}(v(0),0)\\ w(t)=F_{r}(v)(t)=f_{r}(v(t),w(t_{i-1})) \end{array}$$

for *t_i_*_-1_ < *t*< *t_i_*, 1 < *i* < *N*, with

(2) $$f_{r}(v,w)=\max(v-r,\min(v+r,w))$$

where 0<*t*_1_<*t*_2_<···<*t_N_* is a division of the time domain (0 *t_N_*) to ensure that the input signal *v*(*t*) is monotonic within each subinterval (*t_i_*_-1_ *t_i_*). The Play operator have partial memory and rate-independent properties. That is, the output of the Play operator depends not only on current input, but also on the input history. However, the input rate does not change the output shape. As an illustration, Figure 2 shows the input–output relationship of play operator. It is easy to notice that there is a symmetry center in the input–output trajectory.

The PI model utilizes the Play operator *F_r_*(*v*)(*t*) to describe the hysteresis relationship between input *v* and output Γ*_PI_*:

(3) $$\{\begin{array}{l} \Gamma_{PI}(v)(k)=p_{0}v(k)+\sum_{i=1}^{n}p(r_{i})F_{r_{i}}(v)(k)\\ s.t.\;p(r_{i})\geq 0 \end{array}$$

where *n* is the number of Play operators, *p*_0_ is a positive constant. *r_i_* is the threshold of the Play operator, *p*(*r_i_*) represents the weighted coefficient for the threshold *r_i_* and approaches 0 as *r_i_* becomes larger. If *p*(*r_i_*) < 0, the PI model will fail to correctly describe hysteresis minor loops.

**Figure 3:**
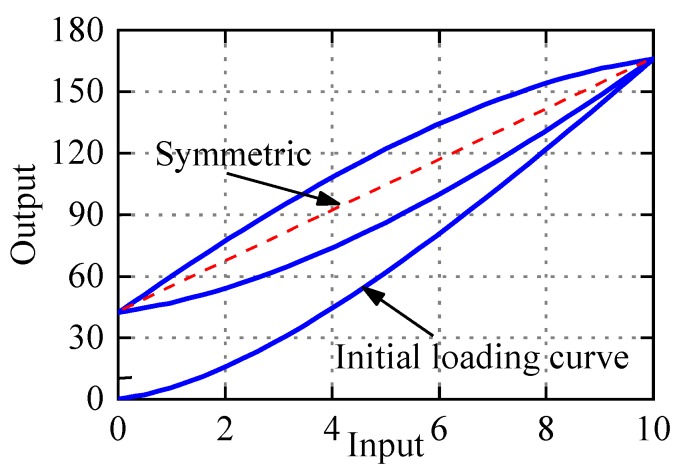
Hysteresis loops generated by the Prandtl–Ishlinskii (PI) model.

Observed from Equation (3), the PI model is composed of the weighted superposition of Play operator *F_r_*(*v*)(*t*) and linear input signal. The input–output curve is parallelogram, which is in central symmetry, so the PI model can only be used to characterize symmetric hysteresis. As an illustration, Figure 3 shows the hysteresis loop generated by the PI model with *p*_0_ = 2.1, *p*(*r_i_*) = 2.6*e*^−0.25*r*^*^i^*, *r_i_* = 0:0.5:9.5, and input *v*(*t*) = 5 + 5sin(2π*t* − π/2). It is obvious that the hysteresis curve generated by PI model is symmetrical. But PEAs often exhibit more or less asymmetric characteristics as shown in Figure 1. In addition, the max displacement of zero voltage in the PI model is uniquely determined by the initial loading curve, and the relationship can be expressed by Equation (4). In fact, the max displacement of the zero voltage is not necessarily related to the initial loading curve, which reflects that the Play operator lacks accuracy and flexibility in describing displacement near zero voltage. One of the motivations of the paper is to modify the PI model to flexibly and accurately represent the asymmetric hysteresis of PEAs.

(4) $$y(0)=\sum_{i=1}^{n}p(r_{i})r_{i}$$

### 2.2. Polynomial-Modified Prandtl–Ishlinskii Model

Utilizing PI model to describe the asymmetric hysteresis of PEAs will produce considerable errors, which cannot meet the needs of precise positioning. To characterize accurately the asymmetric hysteresis, many modified PI models have been proposed, such as Ku-PI model, Gu-PI model, GPI model, and etc. Although these modified PI models can describe the asymmetric hysteresis to some extent, they still have some limitations. To improve the flexibility and accuracy of the model in describing asymmetric hysteresis, we propose Modified-Play (M-Play) operator and polynomial-modified PI model (PMPI).

The M-Play operator is derived from the Play operator, and it is formed by multiplying the threshold value of Play operator on the descending edge by a delay coefficient *η* > −1. The M-Play operator can be written as

(5) $$\left\{ \begin{array}{l} w(0)=F_{r,\eta_{i}}(v)(0)=f_{r,\eta_{i}}(v(0),0)\\ w(t)=F_{r,\eta_{i}}(v)(t)=f_{r,\eta_{i}}(v(0),w(t_{i})) \end{array}\right.$$

where

(6) $$f_{r,\eta_{i}}(v,w)=\max(v-r,\min(v+\eta_{i}r,w))$$

The coefficient *η* alters the threshold of the descending edge. The larger the *η* value, the more obvious the delay in the descending state. With proper values of *η* for every individual M-Play operator, the flexibility and accuracy of the model can be significantly enhanced.

**Figure 4:**
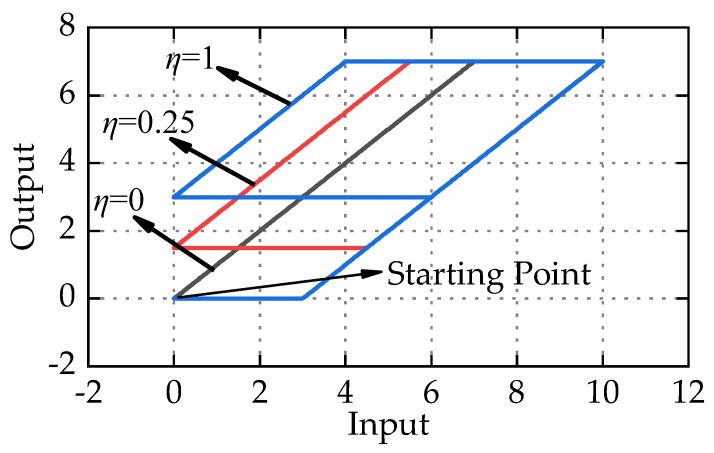
The response of M-Play operators with different delay coefficient *η.*

Figure 4 shows the response of M-Play operators with different delay coefficient *η*. Obviously, one-side Play operator and two-side Play operator are special cases of M-Play operator. When *η* = 0, M-Play operator is equivalent to one-side Play operator, and when *η* = 1, M-Play operator is equivalent to two-side Play operator.

Remarks: From Figure 3, although the delay coefficient *η* is introduced, it can be seen from the input and output trajectories of M-Play operator still exist in the symmetry center. The weighted superposition of M-Play operators alone cannot characterize the asymmetric hysteresis, and the delay coefficient *η* is only used to improve the description of the displacement near zero voltage.

In this paper, the proposed PMPI model is formulated as:

(7) $$\{\begin{array}{l} H(v)(k)=\Gamma_{PI,\eta }(v)(k)+P(v)(k)\\ \Gamma_{PI}(v)(k)=p_{0}v(k)+\sum_{i=1}^{n}p_{i}F_{r_{i},\eta_{i}}(v)(k)\\ P(v)(k)=a_{1}v{\left(k\right)}^{3}+a_{2}v{\left(k\right)}^{2}+a_{3}\\ s.t.\;p_{i}\geq 0,\;\eta_{i}\geq -1 \end{array}$$

where *p*_0_ and *p*(*r_i_*) are defined the same as the ones in PI model (3), and *F_r,__η__i_* are the M-Play operator. A third-degree memoryless polynomial *P*(*v*)(*t*) is used to characterize asymmetric hysteresis of PEAs.

(8) $$y(0)=\sum_{i=1}^{n}p(r_{i})\eta_{i}r_{i}+a_{3}$$

**Figure 5:**
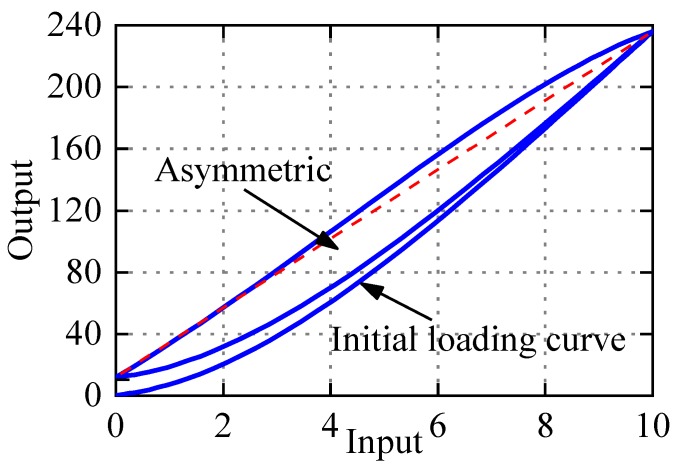
Hysteresis loops generated by polynomial-modified PI (PMPI) model.

The proposed PMPI consists of two parts: several weighted M-Play operators (denoted as MPI model) and memoryless polynomial *P*(*v*)(*t*). The introduction of M-Play operator enables the PMPI model to describe accurately the displacement near zero voltage. It can be seen from Equation (8) that, in PMPI model, the max displacement of zero voltage is adjusted flexibly by the delay coefficient η. The combination of M-Play operators and third-order memoryless polynomial can characterize the asymmetric hysteresis of PEAs. To illustrate the advantages of PMPI model, Figure 5 shows the hysteresis loops generated by PMPI model (7) with *P*(*v*)(*t*) = −0.05*v*^3^ + 1.2*v*^2^ + 0.2 and *η* = 0:0.025:0.475. It is worth noting that weight function *p*(*r*), positive constant *p*_0_, and input *v*(*t*) are the same as of the ones used in the hysteresis loops in Figure 3. In contrast, it is the M-Play operator and memoryless polynomial that enabled the PMPI model to characterize flexibly and accurately the serious asymmetric hysteresis.

### 2.3. Congruency Property

The congruency property is one of the basic properties of hysteresis model. The congruency property means that minor hysteresis loops with different input history are congruent in the same input range. In this section, we prove the congruency property of PMPI model and establish the minor loop mathematical model.

Because of the introduction of M-Play operator and memoryless polynomial *P*(*v*)(*t*), the congruency property of PMPI model needs to be proven. Memoryless polynomial *P*(*v*)(*t*) is a bijective function and has no partial memory, its output depends only on the current input. Therefore, the congruency property of PMPI model depends on the MPI model.

**Figure 6:**
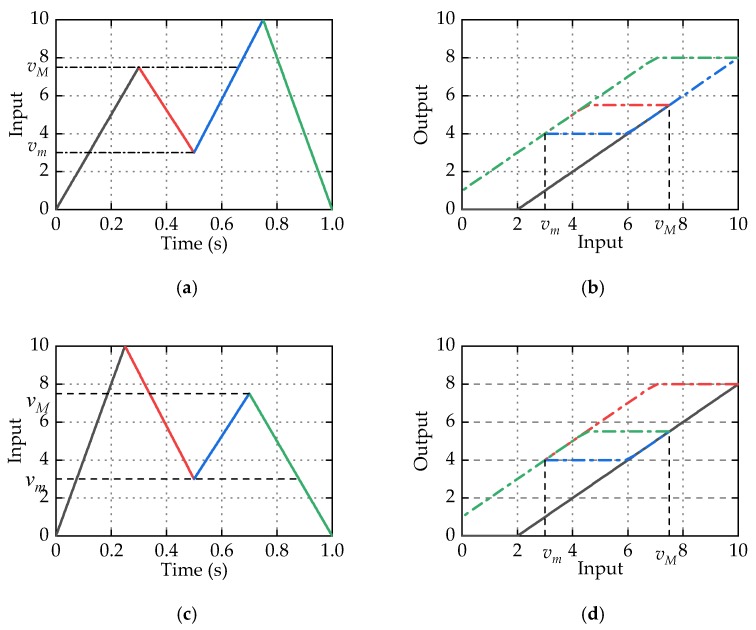
Hysteresis loops of M-Play operator with different input history in the same input range. (**a**) Input history 1; (**b**) hysteresis curve generated by input history 1; (**c**) input history 2; (**d**) hysteresis curve generated by input history 2

To illustrate the congruency property of MPI model, Figure 6 shows the hysteresis loops of M-Play operator with different input history in the same input range. Figure 6a,c shows two input signals with different input history in the same range, and Figure 6b,d shows the corresponding hysteresis curves. Combining these four graphs, it can be clearly seen that, although the input history of the two signals is different, the shapes of the corresponding minor loop formed by M-Play operators are same in the same input range (*v_m_ v_M_*). This example illustrates the congruency property of the M-Play operator, and further demonstrates that MPI model has congruency property.

**Figure 7:**
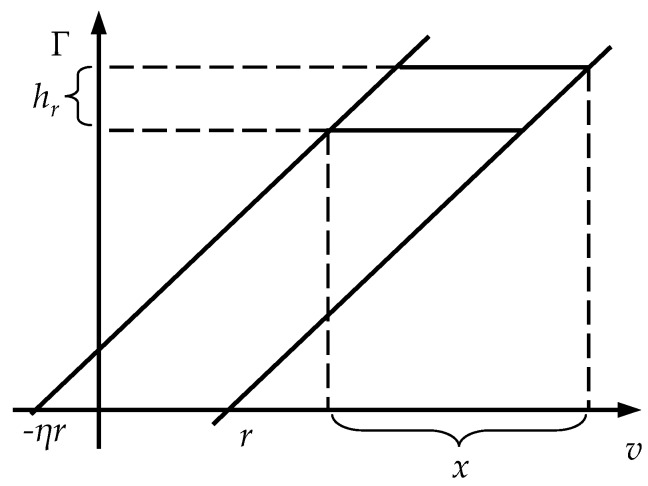
Geometric relations of the minor loop of M-Play operators.

Sprekels’s monograph [27] has proved that the shape of minor loop is uniquely determined when the vertical height *h* of minor loop are constant. Therefore, we quantitatively represent the shape of minor loop with its vertical height *h*. Figure 7 shows the geometric relations of the minor loop of M-Play operator. It can be seen from the figure that the relationship between vertical height *h_r_* and interval length *x* can be formulated as:

(9) $$h_{r}(x)=\max(x-(1+\eta )r,0)$$

where *x* is the interval length of input range (*v_m_ v_M_*). When *x* < (1 + *η*)*r*, the M-Play operator cannot form minor loop, that is, *h_r_* = 0. Based on Equation (8), the vertical height *h_m_* of the minor loop of MPI model can be expressed as:

(10) $$h_{m}(x)=p_{0}x+\sum_{i=1}^{n}p_{i}h_{r_{i}}(x)$$

As mentioned above, the output of memoryless polynomial function *P*(*v*)(*t*) only depends on the current input, and there is no partial memory. The PMPI model is based on the MPI model plus *P*(*v*)(*t*), so the vertical height *h_pm_* of the minor loop of PMPI model can be expressed as:

(11) $$h_{pm}(x)=p_{0}x+\sum_{i=1}^{n}p_{i}h_{r_{i}}(x)+[P(v_{M})-P(v_{m})]$$

**Figure 8:**
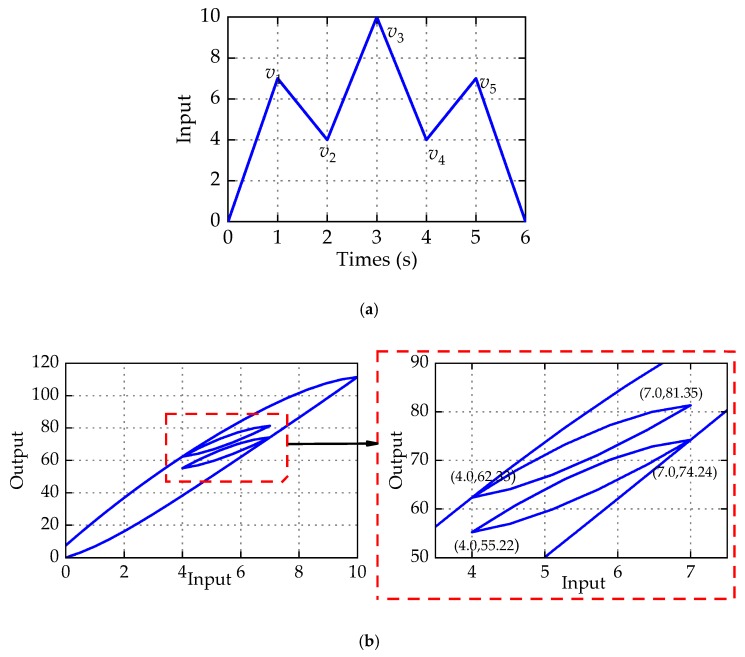
Simulation of the congruency property. (**a**) Input voltage. (**b**) Hysteresis loops.

For example, the input *v*(*t*) is given to verify the correctness of Equation (11). In this case, the sequence of input maxima and minima is defined as {0→7→4→10→4→7→0}. Thus, the input voltage has the same input range (4 7). The parameters of the PMPI model are chosen as *r* = 0:0.5:9.5, *p*_0_ = 3.93, *p_i_* = 2.5*e*^−0.44*ri*^, *η* = 0:0.05:0.95, *P*(*v*)(*t*) = −0.02*v*^3^ − 0.48*v*^2^. For the input voltage shown in Figure 8a, the associated hysteresis loops of PMPI model are given in Figure 8b. It can be seen from the partial enlarged drawing of Figure 8b that the shapes of the minor loops are identical in the same input range and the height of the minor loops are both 19.02 μm. According to the Equation (11) derived above, the vertical height of the minor loops also are 19.02 μm. This example verifies that the PMPI model also has congruency property and the minor loop mathematical model is correct.

## 3. The Design and Analysis of the Inverse Model Compensator

**Figure 9:**
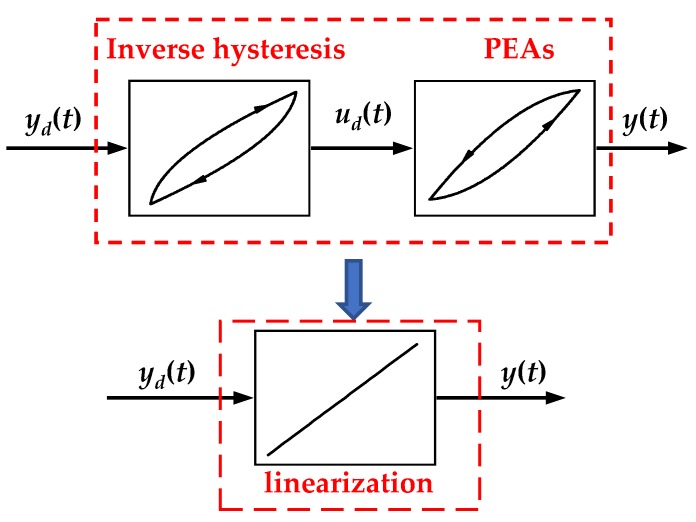
Schematic illustration of feedforward compensation based on inverse hysteresis model.

Feedforward compensation based on inverse hysteresis model is efficient and practical for reducing hysteresis effect in open-loop systems. The main idea of this method is to construct inverse hysteresis model, which is cascaded to the controlled object for feedforward control. After the cascade, the piezoelectric system can be approximated as a linear system, as shown in Figure 9. In this section, based on PMPI model, we will design the inverse model (I-M) compensator to compensate hysteresis and analyze its stability.

### 3.1. Inverse model Compensator Design

The MPI model introduces delay coefficient *η* on the basis of PI model, but this has no influence on the inverse MPI model, that is, the parameter expression of inverse MPI model is the same as that of inverse PI model. This is because that the parameter expression of inverse PI model is derived by the initial loading curve, the delay coefficient *η* only has influence on the descending edge but not the initial loading curve. The initial loading curve is still expressed as:

(12) $$\varphi (r)=p_{0}r+\int_{0}^{r}p(\zeta )(r-\zeta )d\zeta$$

**Figure 10:**
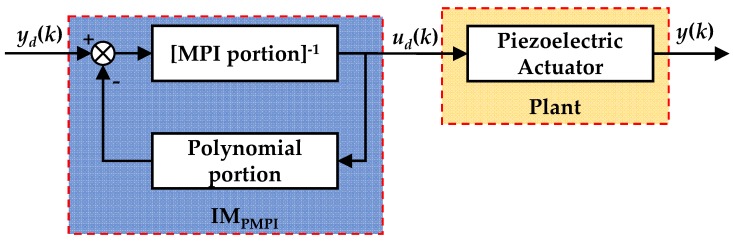
Schematic diagram of inverse model (I-M) compensator.

The MPI model has an analytical inverse model, but the PMPI model has one more memoryless polynomial *P*[*u*](*k*). Hence, there is no analytical inverse model. The inverse PMPI model can only be obtained by iterating the memoryless part. In this section we utilize the iterative structure to design a compensator as shown in Figure 10. In practical applications, most compensator systems are discrete, when the sampling frequency is sufficiently high, *u_i_* ≈ *u_i_*_−1_. So the I-M compensator can be expressed as:

(13) $$u_{d}(k)=H^{-1}[y_{d}](k)=\Gamma_{MPI}^{-1}(y_{d}[k]-P[u_{d}](k-1))$$

where *y_d_*(*k*) is the desired displacement, and *u_d_*(*k*) is the desired control voltage. $\Gamma_{1\;MPI}^{-}$[·](*k*) stands for the inverse MPI model and can be calculated according to the following formula:

(14) $$\Gamma_{MPI}^{-1}[y_{d}](k)={\hat{p}}_{0}y_{d}(k)+\sum_{i=1}^{n}{\hat{p}}_{i}F_{{\hat{r}}_{i},\eta_{i}}[y_{d}](k)$$

with

(15) $$\begin{array}{l} \hat{p}=\frac{1}{p_{0}},\;s.t.\;p_{0}>0\\ {\hat{p}}_{i}=-\frac{p_{i}}{\left(p_{0}+\sum_{j=1}^{i}p_{j})(p_{0}+\sum_{j=1}^{i-1}p_{j}\right)}\\ {\hat{r}}_{i}=p_{0}r_{i}+\sum_{j=1}^{i-1}p_{j}(r_{i}-r_{j}) \end{array}$$

### 3.2. Stability Analysis

It can be seen from Figure 10 and Equation (11) that I-M compensator obtains the desired control voltage *u_d_*(*k*) by iterative solution. The divergence problem may occur during the iterative solution process. Therefore, it is necessary to analyze the stability of the I-M compensator. In this paper, the small gain theorem is used to analyze the stability. The small gain theorem states that when the maximum closed-loop gain of a closed-loop system satisfies |K|<1, then the system is stable in any case. The stable condition can be described as following:

(16) $$\begin{array}{l} \max\left\{K_{inv}(k)|K_{inv}(k)=\left|\frac{d\Gamma_{MPI}^{-1}[y_{d}](k)}{dy_{d}}\cdot \frac{dP[u_{d}](k)}{du_{d}}\right|\right\}<1\\ \Rightarrow p_{0}>\max\left|3a_{1}u_{d}{\left(k\right)}^{2}+2a_{2}u_{d}(k)\right| \end{array}$$

where *K_inv_*[*u*](*k*) denotes the absolute gain of the I-M compensator, and *p*_0_ *a*_1_ and *a*_2_ are the parameters of the PMPI model.

Proof: To satisfy Equation (16), the maximum value of the product of absolute gain |*dP*[*u_d_*](*k*)/*du_d_*(*k*)| and |*d*($\Gamma_{1\;MPI}^{-}$[*y_d_*](*k*)/*d*(*y_d_*(*k*)))| should be less than 1. From Equation (6), we can get

(17) $$0\leq d(F_{{\hat{r}}_{i},\eta_{i}}[y_{d}](k)/d(y_{d}(k)))\leq 1$$

Combining Equations (15), the following relationship can be derived

(18) $$\begin{array}{ll} \sum_{i=1}^{n}{\hat{p}}_{i} & =\sum_{i=1}^{n}\left\{\frac{1}{p_{0}+\sum_{j=1}^{i}p_{j}}-\frac{1}{p_{0}+\sum_{j=1}^{i-1}p_{j}}\right\}\\ & =\frac{1}{p_{0}+\sum_{i=1}^{n}p_{i}}-\frac{1}{p_{0}}=\frac{1}{p_{0}+\sum_{i=1}^{n}p_{i}}-{\hat{p}}_{0}\Rightarrow -{\hat{p}}_{0}<\sum_{i=1}^{n}{\hat{p}}_{i}<0 \end{array}$$

Therefore, the upper limit of the gain *d*(

$$\Gamma_{1\;MPI}^{-}$$

[*y_d_*](*k*)/*d*(*y_d_*(*k*))) is 1/*p*_0_. Since the memoryless polynomial *P*[*u*](*k*) is differentiable, its gain can be expressed by the following formula:

(19) $$\left|dP[u_{d}](k)/du_{d}\right|=\left|3a_{1}u_{d}{\left(k\right)}^{2}+2a_{2}u_{d}(k)\right|$$

Combining Equations (18) and (19), it can be concluded that when the identified parameters of PMPI model meet the condition (16), I-M compensator is globally stable.

The I-M compensator using iterative structure has superior performance in accuracy and response speed, but it may appear that the parameter identified by PMPI model does not satisfy Equation (16). Once this happens, the proportional gain *k_e_u_d_*(*k*) addressed in Equation (20) can be introduced to adjust the ratio between the MPI and memoryless polynomial part. In addition, the proportional gain *k_e_u_d_*(*k*) can greatly improve the convergence speed of I-M compensator. Compared with the dichotomy of pure iterative, the I-M compensator utilizes the characteristic of the analytical inverse of the MPI model, and its iterative process is approximately open-loop. Hence, the I-M compensator has fewer iterative steps, faster convergence speed, and higher accuracy.

(20) $$\begin{array}{l} H[u_{d}](k)=(\Gamma_{MPI}[u_{d}](k)+k_{e}u_{d}(k))+(P[u_{d}](k)-k_{e}u_{d}(k))\\ \Rightarrow p_{0}+k_{e}>\max\left|3a_{1}u{\left(k\right)}^{2}+2a_{2}u(k)-k_{e}\right| \end{array}$$

## 4. Experimental Verification and Discussion

In this section, an experimental platform is established. The experiment is conducted to verify the effectiveness of the PMPI model and I-M compensator in hysteresis modeling and compensation.

### 4.1. Experimental Setup

**Figure 11:**
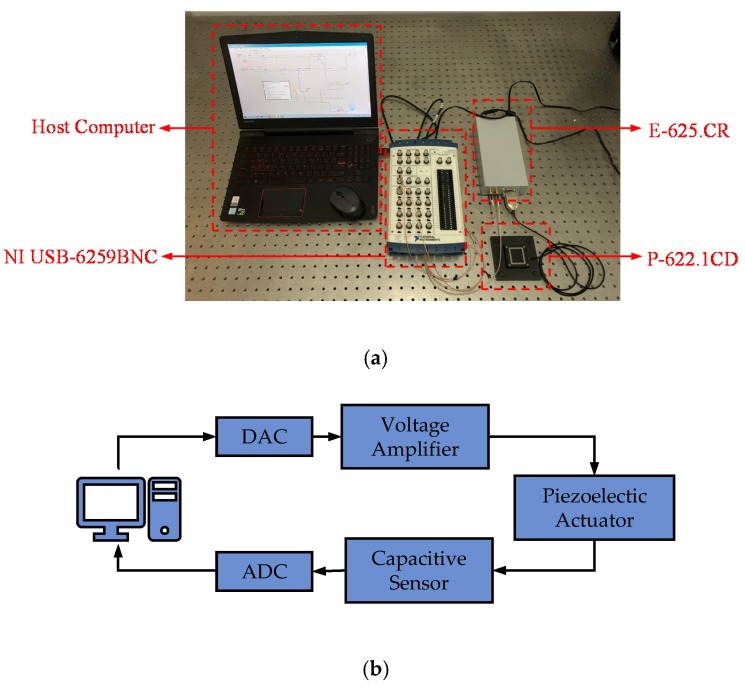
Experimental system. (**a**) Experimental platform; (**b**) process block diagram.

As shown in Figure 11a, the experimental platform consists of a computer, a USB-6259BNC (from National Instruments, Austin, TX, USA) data acquisition card, a 1-D piezoelectric micro-motion platform, and a piezoelectric servo controller E-625.CR (from Piezomechanik, München, Germany). The P-622.1CD (from Piezomechanik, München, Germany) has a maximum stroke of 200 μm and a built-in capacitive displacement sensor. The E-625.CR has a piezoelectric amplifier and displacement acquisition module. Its voltage amplification factor is 10 and the sensitivity of the displacement acquisition module is 20 μm/V. The USB-6259BNC has multiple 16-bit digital-to-analog converters and 16-bit analog-to-digital converters and cooperates with the host computer to realize the real-time control of the micro-motion platform. Figure 11b shows the process block diagram of the experimental system.

### 4.2. Asymmetric Hysteresis Description Results and Discussion

To experimentally validate the PMPI model, the first step is to identify the parameters of PMPI model. Model type and parameter identification both affect the accuracy of hysteretic modeling. Many identification algorithms [28,29,30] have been proposed to obtain model parameters, such as least square method (LSE), particle swarm optimization (PSO), and differential evolution (DE) algorithm. However, ensuring that the identified parameters are the global optimal solutions is a challenging task. In this section, the hybrid algorithm Nelder–Mead differential evolution (NM-DE) [31], based on differential evolution and simplex algorithm, is used to identify the parameters of the PMPI model. The NM-DE algorithm takes into account both global and local search capabilities, and has the advantages of fast convergence and high accuracy.

It should be noted that the larger the number of operators, the more accurately the model can describe the hysteresis in theory. Table 1 shows the relationship between the number of operators n, identification errors, and run time, where the runtime reflects indirectly the computation. From this Table, it can be observed that modest increase in the number of operator can improve the accuracy of the model, but further increase in the number of operator show no significant improvement in the accuracy of model, the identification errors are almost at the same level when n = 10,20,30. In addition, increase in the number of operators will increase the run time (computation) which further affects the real time performance of compensation. We select *n* = 10 for the case studies. As mentioned above, the weighting coefficient *p*(*r_i_*) approaches 0 as *r_i_* becomes larger. The weighting coefficient *p*(*r_i_*) can be expressed as *p*(*r_i_*) = *α*_1_*e*^−*α*_2_^^*r*_i_^. This form reduces the number of parameters to be identified and greatly reduces the identification burden.

**Figure 12:**
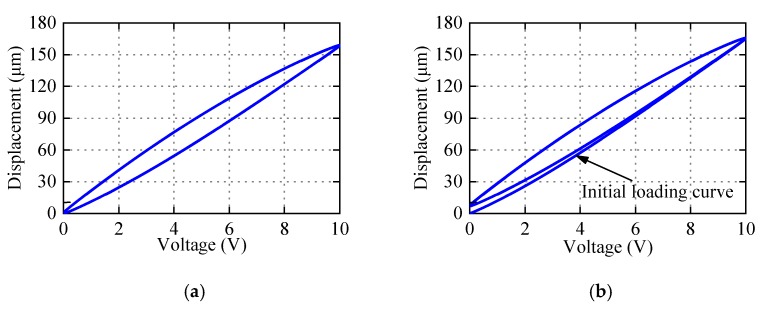
Two cases of comparative experiment. (**a**) Case 1: the initial loading curve is not considered; (**b**) Case 2: the initial loading curve is considered.

**Figure 13:**
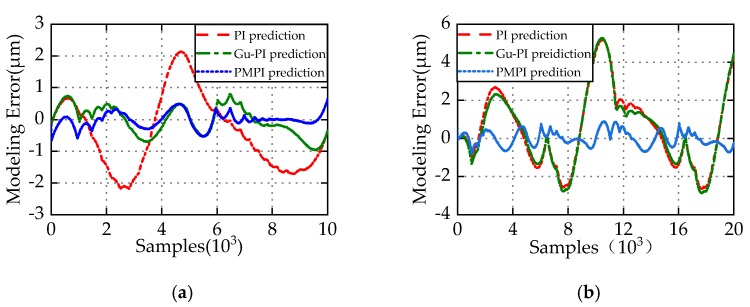
Performance comparison of three modeling methods in two cases. (**a**) Modeling errors in Case 1; (**b**) modeling errors in Case 2.

To demonstrate the superiority of PMPI model in characterizing asymmetric hysteresis, comparison of the three models PI, Gu-PI, and PMPI was carried out. The number of operators *n* is set to be 10, the thresholds are the same, and the parameters of models are the optimal values obtained after repeated identifications. The comparison experiments were carried out respectively in two cases (Case 1 and Case 2) as shown in Figure 12. The input–output curves of the three models appear to coincide because of the small modeling error. To directly reflect the superiority of PMPI model in hysteresis modeling accuracy, Figure 13a,b show respectively the modeling errors of the three models PI, Gu-PI, PMPI in two cases. In order to evaluate the accuracy of hysteresis model and quantify the modelling error, the maximum absolute error (MAE), the mean absolute deviation (MAD), and the root-mean-square error (RMSE) are defined as follows.

(21) $$\{\begin{array}{l} \mathrm{MAE}=\underset{1\leq i\leq N}{\max}\left|\hat{y}(i)-y(i)\right|\\ \mathrm{MRE}=\frac{MAE}{y_{\max}}\times 100\%\\ \mathrm{MAD}=\frac{1}{N}\sum_{i=1}^{N}\left|\hat{y}(i)-y(i)\right|\\ \mathrm{RMSE}=\sqrt{\frac{1}{N}\sum_{i=1}^{N}{\left[\hat{y}(i)-y(i)\right]}^{2}} \end{array}$$

where *N* is the number of samples, *y*(*i*) is the real measured displacement, *y*(*i*) is the model predicted displacement, and *y*_max_ is the maximum measured displacement. Among them, the MAE and MRE are used to evaluate local accuracy, and the MAD and RMSE are used to evaluate global accuracy.

The modeling error evaluation results of the three models in two cases are respectively listed in Table 2 and Table 3. It can be seen from Table 2 that the prediction errors of the Gu-PI and PMPI model are significantly lower than that of the PI model in Case 1, and the MRE of prediction are only 0.968% and 0.698%. The result shows that the Gu-PI and PMPI model have obvious advantages in characterizing asymmetric hysteresis in Case 1. However, compared with PI and Gu-PI model, the accuracy of the PMPI model is significantly improved in Case 2, and the MAE of prediction is reduced by 83.3%. This is due to the lack of accuracy of Play operator in describing the displacement near zero voltage on the descending edge. This deficiency of Play operator shows that the local accuracy of Gu-PI model is approximately equal to PI model. The M-Play operator significantly improves the flexibility and accuracy of PMPI model. If the initial loading curve is not considered in hysteresis compensation, the compensator must make PEAs run for a period of time in advance, which will undoubtedly increase the burden of the compensator. In summary, Case 1 has high modeling accuracy, but it will increase the burden of the compensator. Case 2 has slightly low modeling accuracy, but the compensator has no such concern. The proposed PMPI model has superior modeling ability for hysteresis asymmetry in both cases.

### 4.3. Hysteresis Compensation Results and Discussion

**Figure 14:**
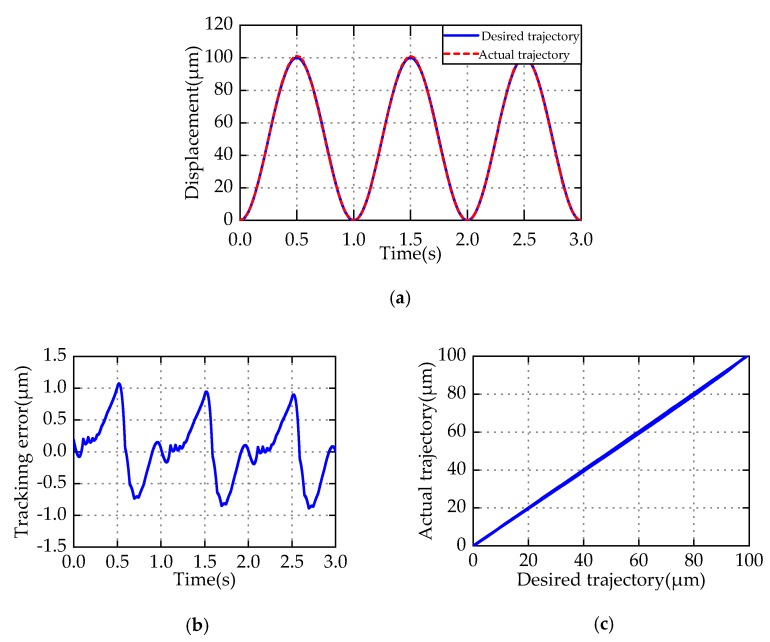
Periodic sinusoidal reference tracking experiment. (**a**) Trajectory tracking; (**b**) tracking error; (**c**) the relationship between desired and actual displacement.

Table 4 lists the identified parameters of the PMPI model, the parameters of PMPI model satisfy the condition (14) in the range (0 10). Therefore, the I-M compensator is globally stable. To verify the effectiveness of the I-M compensator, the tracking experiment with periodic sinusoidal references with *y_r_* = 50 + 50sin(2πt−π/2) is conducted. Figure 14a shows the comparison of the desired and actual trajectory. After compensation, the actual displacement can track the desired trajectory well, and no tracking loss occurs. Figure 14b shows the tracking errors, defined as the difference between the desired and actual trajectory. The MAE is 1.07 μm, the MRE is 1.07%, and the MAD is less than 0.4 μm. It is worth mentioning that, because of the existence of modeling uncertainty, the tracking errors appear periodic in periodic tracking experiments, which can be seen as systematic error, which can be eliminated by closed-loop control. To more intuitively reflect the compensation effect, Figure 14c shows the relationship between the desired and actual displacements. After compensation, the input–output shows an approximate linear relationship. The error is one order of magnitude less than that without any control, which shows that the I-M compensator can well suppress the hysteresis characteristics of PEAs.

**Figure 15:**
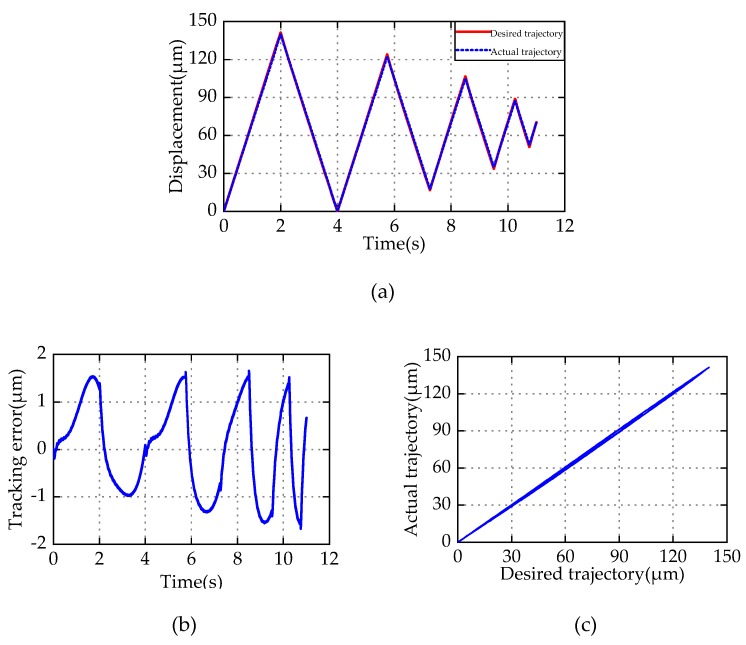
Frequency conversion attenuated triangular wave reference tracking experiment. (**a**) Trajectory tracking; (**b**) tracking error; (**c**) relationship between desired and actual displacement.

To further verify the effectiveness of I-M compensator, a tracking experiment of frequency conversion attenuated triangular wave is performed. Figure 15 shows the results of this tracking experiment. It can be seen that the I-M compensator still has good tracking performance in tracking complex trajectory. The MRE is 1.18%, which is slightly larger than the ones of periodic sinusoidal. The experimental result further demonstrates the effectiveness of the I-M controller in hysteresis compensation.

## 5. Conclusions

Because of its simple structure and analytical inverse, the PI model are used widely in hysteretic nonlinear modeling and compensation. However, the PI model can only describe symmetric hysteresis. In this paper, based on PI model, we provide a novel PMPI model to describe and compensate asymmetric hysteresis of PEAs. First, the PMPI model is introduced, which includes the M-Play operator and memoryless polynomial. Then, the congruency property of PMPI model is analyzed and verified. The minor loop mathematical model is also established. Next, the correctness of the PMPI model is proved by simulation. It should be noted that when considering the initial loading curve, the PMPI model can accurately characterize asymmetric hysteresis. Compared with the PI model and the Gu-PI model, the error of PMPI model is reduced by 83.3%. In the end, based on the PMPI model, the I-M compensator is designed for hysteresis compensation. The stability of I-M compensator is analyzed. The experimental results show that the I-M controller has superior tracking performance.

Although the PMPI model has satisfactory results for asymmetric hysteresis, it is not suitable for rate-dependent and load-dependent hysteresis. In the future, further research is to expand the PMPI model to rate-dependent and load-dependent applications.

## Figures and Tables

**Table 1:** The relationship between the number of operators *n*, identification error, and run time.

Number of Operators *n*	Identification Error (μm)	Run Time (ms)
5	1.503	48.93
10	0.884	53.30
20	0.831	69.49
30	0.848	87.50

**Table 2:** Comparison of three model errors in Case 1.

Model	MAE (μm)	MRE (%)	MAD (μm)	RMSE (μm)
PI	2.186	1.37	1.058	1.243
Gu-PI	0.968	0.61	0.397	0.463
PMPI	0.698	0.44	0.172	0.232

**Table 3:** Comparison of three model errors in Case 2.

Model	MAE (μm)	MRE (%)	MAD (μm)	RMSE (μm)
PI	5.193	3.14	1.654	2.049
Gu-PI	5.280	3.19	1.627	2.038
PMPI	0.905	0.55	0.334	0.397

**Table 4:** The identified parameters of PMPI model.

i	r_i_	α_i_	η_i_	a_i_
1	0	6.904	0.849	0.037
2	1	0.517	0.043	−0.647
3	2	-	0.276	0
4	3		0.376	-
5	4		0.336	
6	5		0.443	
7	6		0.561	
8	7		0.335	
9	8		0.158	
10	9		0.040	
*p*_0_	4.770		-	
